# Cangfu Daotan Wan alleviates polycystic ovary syndrome with phlegm-dampness syndrome via disruption of the PKP3/ERCC1/MAPK axis

**DOI:** 10.1186/s13048-023-01200-7

**Published:** 2023-07-07

**Authors:** Yuan Li, Haicui Wu, Ying Guo, Chaofeng Wei, Lu Guan, Wenhan Ju, Fang Lian

**Affiliations:** 1 Department of Reproduction and Genetics, Affiliated Hospital of Shandong University of Traditional Chinese Medicine, No. 42, Wenhua West Road, Jinan, 250011 Shandong Province P. R. China; 2 Shandong University of Traditional Chinese Medicine, Jinan, 250355 P. R. China; 3 The First Clinical Medical College, Shandong University of Traditional Chinese Medicine, Jinan, 250355 P. R. China

**Keywords:** Polycystic ovary syndrome, Cangfu daotan wan, Ovarian granulosa cells, DNA methylation, PKP3, MAPK pathway, ERCC1

## Abstract

**Background/Aim:**

Cangfu Daotan Wan (CFDTW) has been widely used for polycystic ovary syndrome (PCOS) patients in the type of stagnation of phlegm and dampness. In this study, we aimed to evaluate the mechanism underlying the therapeutic effect of CFDTW on PCOS with phlegm-dampness syndrome (PDS).

**Methods:**

*In silico* analysis was adopted to identify CFDTW potential targets and the downstream pathways in the treatment of PCOS. Expression of PKP3 was examined in the ovarian granulosa cells from PCOS patients with PDS and rat PCOS models induced by dehydroepiandrosterone (DHEA). PKP3/ERCC1 was overexpressed or underexpressed or combined with CFDTW treatment in ovarian granulosa cells to assay the effect of CFDTW on ovarian granulosa cell functions *via* the PKP3/MAPK/ERCC1 axis.

**Results:**

Clinical samples and ovarian granulosa cells of rat models were characterized by hypomethylated PKP3 promoter and upregulated PKP3 expression. CFDTW reduced PKP3 expression by enhancing the methylation of PKP3 promoter, leading to proliferation of ovarian granulosa cells, increasing S and G2/M phase-arrested cells, and arresting their apoptosis. PKP3 augmented ERCC1 expression by activating the MAPK pathway. In addition, CFDTW facilitated the proliferation of ovarian granulosa cells and repressed their apoptosis by regulating PKP3/MAPK/ERCC1 axis.

**Conclusion:**

Taken together, this study illuminates how CFDTW confers therapeutic effects on PCOS patients with PDS, which may offer a novel theranostic marker in PCOS.

**Supplementary Information:**

The online version contains supplementary material available at 10.1186/s13048-023-01200-7.

## Background

Polycystic ovary syndrome (PCOS), defined as combined symptoms of androgen excess and abnormal functions in ovarian, is a commonly occurring endocrine and metabolic disease among premenopausal women [1]. Women with PCOS exhibit an increased risk of type 2 diabetes mellitus, ovarian malignancy, hypertension, vascular thrombosis, hepatic steatosis and metabolic syndrome, cerebrovascular and cardiovascular events, and psychosexual disorders [2]. Traditional Chinese medicine believes that the abnormal changes in qi, blood, and body fluid initiates phlegm and dampness, and specifically, accumulation of phlegm exacerbates the dysfunction of qi, blood, and body fluid; phlegm-dampness represents the predominant pathological basis of PCOS and reducing phlegm and dampness is the major management principle [3, 4].

Cangfu Daotan Wan (CFDTW) is a type of traditional Chinese medicine and represents one of the most common prescriptions applied for PCOS patients with phlegm-dampness syndrome (PDS) due to its effects of removing the phlegm and dampness [5, 6]. PKP3 is a member of the armadillo protein family essential for the maintenance of cell-cell adhesion, the dysregulation of which is responsible for inherited diseases and cancer pathogenesis [7]. PKP3 shows upregulation in ovarian cancer tissues and its expression serves as a prognostic biomarker for ovarian cancer patients [8]. In addition, enforced PKP3 expression is capable of facilitating the proliferation, formation, and invasion of ovarian cancer cells, which shares correlation with the activated MAPK pathway [9, 10]. MAPK is known as a key pathway that modulates diverse cellular processes, including proliferation, differentiation, apoptosis, and stress responses [11]. MAPK has been reported to be associated with endometrial related reproductive disorders and has higher activity in patients with PCOS [12]. Activation of the MAPK pathway participates in the pathogenesis of PCOS in rat models induced by dehydroepiandrosterone (DHEA) [13]. The expression of ERCC1 is regulated by the MAPK signaling pathway [14]. Furthermore, enhancement of the MAPK pathway can lead to upregulation of ERCC1, which is a DNA repair gene and is prominently expressed in chemo- or radio-resistant cancers [14, 15]. The expression of ERCC1 is significantly elevated in ovarian cancer tissues and can serve as a critical indicator to judge the severity of ovarian cancer [16]. The above discussion revealed a possible network among CFDTW, PKP3, MAPK and ERCC1 in the progression of PCOS patients with PDS. Herein, the present work was conducted in a bid to reveal the specific mechanism of CFDTW/PKP3/MAPK/ERCC1 axis in PCOS patients with PDS.

## Methods

### Ethics statement

Our work was approved by the Ethics Committee of Affiliated Hospital of Shandong University of Traditional Chinese Medicine (2022022) and implemented in line with the *Declaration of Helsinki*. Informed consent documentation was provided by all subjects before sample collection. Animal experiments were ratified by the Animal Ethics Committee of Affiliated Hospital of Shandong University of Traditional Chinese Medicine (2022044).

### Clinical sample collection

This study recruited 12 PCOS patients with PDS and 6 healthy women who underwent in vitro fertilization (IVF) as the control. Their ovarian granulosa cells were extracted for sequencing. The identification of PCOS with PDS referred to the “Diagnosis and Efficacy Criteria for Diseases and Syndromes of Traditional Chinese Medicine” and the seventh edition of the textbook “Gynecology of Traditional Chinese Medicine”. Two associate senior experts conducted independent syndrome differentiation on the recruited subjects, which were included when the two experts gave a dialectical agreement. The proposed diagnostic criteria of syndrome differentiation of PDS were: main symptoms contained delayed or gradual amenorrhea, a small amount of menstrual period with light color, or long-term marriage infertility; concomitant symptoms contained obesity or mental fatigue and drowsiness; the fullness of chest and abdomen; easy stool or diarrhea during menstruation; a large amount of discharge; tongue and pulse: light, fat, and toothed tongue, greasy moss, and pulse sinking or slippery slow. All patients were diagnosed with the above three main symptoms and were required to meet one or two concomitant symptoms with/without the tongue symptoms.

Twelve patients were grouped into a placebo group and a CFDTW group (n = 6 cases/per group) on the basis of the random number table method. CFDTW was composed of Atractylodes rhizome, Rhizoma Cyperi, Pinellia ternata, Tangerine peel, Shenqu, Dannanxing, Poria cocos, Fructus aurantii, ginger, and tangerine peel, provided by the preparation room of the Affiliated Hospital of Shandong University of Traditional Chinese Medicine, 3 g/pack, 12 bags/bag. CFDTW was taken 3 bags at a time, twice a day. The placebo was prepared by the preparation room of the Affiliated Hospital of Shandong University of Traditional Chinese Medicine, mainly composed of dextrin, 3 g/bag, 12 bags/bag, with 3 bags at a time, twice a day.

PCOS patients with PDS syndrome adopted the standard long-term regimen: oral Diane-35 on the 3rd day of the menstrual period before IVF, 1 tablet a day for a total of 21 tablets, and oral CFDTW or placebo particles until the day of injection with human chorionic gonadotropin (hCG).

On the 21st day of the menstrual period before IVF, if there was no cyst in the vaginal B-ultrasound, a single intramuscular injection of 1.0 mg of GnRHa was given for downregulation. After 14 days, blood was drawn to detect the serum levels of follicle stimulating hormone (FSH), luteinnizing hormone (LH) and estradiol (E2). The endometrial thickness and follicle condition were examined by the vaginal ultrasound to evaluate whether the pituitary downregulation standard met (follicle diameter < 5 mm, endometrial thickness ≤ 5 mm, E2 < 30 pg/mL, FSH < 5 mIU/mL, LH < 5 mIU/mL). If downregulation was successful, an appropriate amount of r-FSH was subcutaneously injected for controlled ovarian hyperstimulation (COH) according to the patients’ age, body mass index, antral follicle count, and basic FSH value. In addition, an appropriate amount of human menopausal gonadotropin (HMG) intramuscular injection was conducted based on the patients’ specific conditions.

### Follicular granulosa cell separation and extraction

Follicular development and serum hormone levels were regularly monitored by B-ultrasound. When there were three or more follicles with an average diameter of ≥ 17 mm in the B-ultrasound, the subject was injected with HCG 10,000 IU intramuscularly combined with E2 level. After about 36 h, follicles were removed. The follicular fluid that dominated the follicle with a diameter of 1.8–2.0 cm was collected. A 16 G puncture needle (Cook) was used to aspirate follicles under the guidance of a transvaginal ultrasound probe, and the dominant follicles with a bilateral ovarian diameter ≥ 18 mm should be punctured at the beginning of the operation, with the blood avoided and follicular fluid retained. The follicle fluid was immediately placed in a sharp-bottomed centrifuge tube at room temperature for centrifugation at 1000 g for 5 min. Following supernatant removal, the pellet (containing granular cells and blood cells) at the tube bottom was mixed with 5 mL of PBS. Another centrifuge tube was supplemented with 5 mL human peripheral blood lymphocyte separation solution, which was then added with the mixture of the pellet and PBS, and centrifuged at 800 g for 20 min. There was a white cell layer between the mixture surface and lymphocyte separation liquid, that is, the granular cells following the removal of red blood cells. The flocculent granular cells floating between the two liquid surfaces were removed, added with PBS, centrifuged at 1000 g for 5 min and then transferred to a 1.5 mL tipped sterile centrifuge tube for another centrifugation at 1500 g for 5 min. The upper layer of liquid was discarded and the pellet was stored in a -80 °C freezer for later use.

### Reduced representation bisulfite sequencing (RRBS)

DNeasy Blood&Tissue Kit (Qiagen) was used for this experiment. Degradation and contamination of genomic DNA was detected using 0.8% agarose gel electrophoresis, and DNA purity was detected using a NanoPhotometer® spectrophotometer (IMPLEN, CA, USA). DNA concentration was detected using the Qubit® DNA Assay Kit in the Qubit® 2.0 Flurometer (Life Technologies, CA, USA). Qualified DNA is collected for future use.

Library construction: a total of 5.2 µg of genomic DNA was supplemented with 26 ng of lambda DNA, which was sonicated to 200–300 bp using a Covaris S220, followed by end repair and adenylation. Cytosine-methylated barcodes were attached to sonicated DNA according to the manufacturer’s instructions. These DNA fragments were treated twice with the Bisulfite EZ DNA Methylation-goldtm Kit (Zymo Research) and single-stranded DNA fragments were subjected to PCR amplification using KAPA HiFi HotStart Uracil + ReadyMix (2X). Library concentration was determined using a Qubit® 2.0 Flurometer (Life Technologies, CA, USA) and quantitative PCR, and the size of insert fragments was determined using an Agilent Bioanalyzer 2100 system.

The constructed library was sequenced using Illumina Hiseq 2500/4000 or Novaseq platform to obtain 125 bp/150 bp paired-end reads. Image analysis and base calling were performed using the Illumina CASAVA pipeline, and finally 125 bp/150 bp paired-end reads were generated.

FastQC (fastqc_v0.11.5) was used to perform basic quality statistics on raw reads. The rasds sequences in FASTQ format generated by the Illumina pipleline were preprocessed with Trimmomati (Trimmomatic-0.36) software, and the parameters were set as: SLIDINGWINDOW: 4:15; LEADING: 3, TRAILING: 3; ILLUMINALIP: adapter.fa: 2: 30: 10; MINLEN:36. The remaining reads that passed all filtering steps were identified as clean reads, and based on this, subsequent analysis was performed. Finally, basic quality control of cleandata reads was performed using FastQC.

Bisulfite-treated reads were calibrated based on the reference genome (-X 700 --dovetail) using Bismark (version 0.16.3; Krueger F, 2011) software to obtain the unique and best alignment. After comparison with the normal genome sequence, the methylation status of all cytosine positions in the reads could be inferred. Identical reads aligned to the same region of the genome are considered duplicates. Sequencing depth and coverage were summarized using deduplication.

IGV browser was used to convert the results of the methylation extractor (bismark_methylation_extractor, --no_overlap) to bigWig format for visualization. The non-coversion rate of sodium bisulfite was calculated as the percentage of cytosines at the cytosine reference site in the lambda genome.

To identify methylation sites, we modeled the sum of methylation counts mC as a binomial (Bin) random variable with a methylation rate r: mC ~ Bln (mC + umC * r). To calculate the methylation level of this sequence, we divided the sequence into multiple bins with a bin size of 10 kb. The sum of methylated and unmethylated read counts in each window was calculated. The methylation level (ML) of each window or C site shows the proportion of methylated Cs and is defined as: ML(C) = reads(mC)/{reads(mC) + reads(C)}. The calculated ML was further corrected for the bisulfite non-conversion ratio according to a previous study [17]. Given the bisulfite non-conversion ratio r, the corrected ML is estimated as: ML (corrected) = (ML-r)/(1-r).

Differentially methylated regions were identified using DSS software [18–20] for subsequent analysis.

### Microarray-based gene expression profiling

Based on the differential methylation analysis results of the RRBS data in the normal, PCOS and PCOS + CFDTW groups, the jvenn tool was applied to identify the overlapping genelist between the normal group and the PCOS group, the PCOS group and the PCOS + CFDTW group, to predict the methylation level of key factors affecting PCOS and the factor methylation level before and after CFDTW treatment. Based on the distribution of DMR on the genome, functional enrichment analysis was implemented on the overlapping genes in the gene body region and the DMR. PCOS-related gene expression datasets GSE8157 and GSE34526 were retrieved from the Gene Expression Omnibus database. GSE8157 dataset consisted of 13 control samples and 10 PCOS samples, while GSE34526 dataset consisted of 3 control samples and 7 PCOS samples. Differential analysis was implemented employing R language “limma” package with |Log_2_FC| > 1 and *p* < 0.05 as the threshold to screen differentially expressed genes. The BATMAN software was adopted to predict the potential targets (Score ≥ 20 and adjusted *p* value ≥ 0.05) of CFDTW for the treatment of PCOS and the related pathways involved.

### Rat PCOS model construction

Twenty-four female Sprague Dawley (SD) rats (aged 21–23 days; Vital River Laboratory Animal Technology Co., Ltd., Beijing, China) were housed individually in a SPF animal laboratory at 22–25 °C with 60–65% humidity under a 12-h light/dark cycle (eat and drink freely). The experiment was started after one week of acclimation. Each group contains eight rats. The rats were continuously injected with DHEA at a dose of 6 mg/100 g body weight (dissolved in 0.2 mL injection oil) for a total of 30 days. During this period, the rats in the control group were only injected with 0.2 mL of injection oil. On the 30th day after DHEA injection, the rats did not ovulate, and the appearance of keratinocytes in the vaginal smear was an indicative of successful model construction.

24 h post successful modeling, rats in the PCOS + CFDTW group received intragastric administration of 6 mg/g CFDTW once a day for 14 days. Rats in the PCOS group received intragastric administration of the same amount of normal saline. Normal rats served as controls.

### Estrous cycle identification

Vaginal smear method was used to identify the estrous cycle of rats. A small amount of normal saline was sucked by a pipette into the rats’ vagina, and mechanically dissociated several times. The vaginal fluid was sucked out and air-dried. The air-dried smear was stained with Giemsa stain (C0133, Beyotime Biotechnology Co., Shanghai, China), punched, dried, and observed under a microscope.

As shown in Figure S1, during proestrus, there were large number of round nucleated epithelial cells, a few white blood cells, and a few anucleated keratinized epithelial cells. During estrus, there appeared almost all large, flat, non-nucleated keratinocytes. In the diestrus, large number of keratinized epithelial cells, and a small number of white blood cells and nucleated epithelial cells gathered in a pile. During the metestrus, there existed a large number of white blood cells, a small number of round nucleated epithelial cells. The red arrows represent nucleated epithelial cells, the green arrows represent keratinized squamous epithelial cells, and the black arrows represent white blood cells.

### Immunohistochemistry

Ovarian tissue samples were immunostained with anti-PKP3 antibody (18338-1-AP, 1 :100, Proteintech ProteinTech Group, Chicago, IL, USA) overnight at 4 °C. The next day, the samples were incubated with secondary antibody for 1 h at ambient temperature, and developed using 3,3’-diaminobenzidine kit (Invitrogen Inc., Carlsbad, CA, USA).

### Hematoxylin-eosin (HE) staining

HE staining kit (C0105, Beyotime) was adopted here. Briefly, ovarian tissue sections were dewaxed twice with xylene for 5–10 min each, rehydrated in descending series of alcohol (100% for 5 min, 90, 80 and 70%, for 2 min each), and rinsed with distilled water for 2 min. Next, the sections were stained with hematoxylin for 5–10 min, counterstained with eosin for 30 s − 2 min, dehydrated in ascending series of alcohol (70, 80, 90 and 100%, 10 s each), cleared twice with xylene for 5 min each, and mounted with neutral gum. The sections were finally observed under an inverted microscope (IX73, Olympus Optical Co., Ltd, Tokyo, Japan).

### Determination of serum hormones in rats

After the last administration, the rats were deprived of food and water for 12 h. Blood was then collected from the heart, left to stand at 4 °C for 1 h, and centrifuged at 3000 g for 10 min to separate the serum. Serum levels of oestradiol (E2; H102-1, Nanjing Jiancheng Bioengineering Institute, Nanjing, China), testosterone (T; H090-1-1, Jiancheng), luteinising hormone (LH; H206-1, Jiancheng), and follicle stimulating hormone (FSH; H101-1, Jiancheng) were assayed utilizing enzyme-linked immunosorbent assay (ELISA) Kit.

### Methylation specific polymerase chain reaction (MSP) and bisulfite genome sequencing (BSP)

Mammalian genomic DNA extraction kit (D0061, Beyotime) was used to extract DNA from rat ovarian tissues, and 21 µg of DNA was collected for bisulfite conversion using rapid bisulfite conversion kit (59,824, Qiagen, Germany), and denatured with 3 M NaOH at 37 °C for 10 min, and at 50 °C with sodium sulfite hydrogen salt for 16 h.

For MSP assay, 0.25 µL Hot-StarTaq Master Mix (203,443, Qiagen), 2 µg bisulfate-treated DNA template, and 0.5 µM primers were added to the 20 µL system for PCR amplification. Afterwards, 20 µL product was separated on a 3% agarose gel, observed, and photographed using the iBright FL1500 imaging system (Invitrogen).

For BSP assay, genomic DNA treated with CpG methyltransferase (Sss I) (New England BioLabs, Inc, Beverly, MA) was used as a positive control, and blank water as a negative control (NC). Primers were designed to recognize the DNA converted by sodium bisulfite and cover the MSP region. The target fragment of the PCR product was purified with a fragment size of 201 bp, and then cloned into the pGEM-T vector (Promega, San Luis Obispo, CA), and 10 clones of each specimen were sequenced by fluorescence-based automated DNA sequencing. The sequence of primers used is depicted in Table S1.

### Isolation, identification, and lentiviral vector transduction of rat ovarian granulosa cells

Female SD rats (21–25 days) were subcutaneously injected with 40 IU of pregnant mare serum gonadotropin. After 48 h, the rats were euthanized by carbon dioxide inhalation, and the ovaries were collected aseptically. The ovarian sac and surrounding fat tissues were removed under a microscope and placed in pre-chilled serum-free DMEM/F12. The follicles were punctured using a 25-gauge needle and filtered, followed by centrifugation at 400 g for 8 min. With the supernatant removal, the cells were collected and cultured in DMEM/F12 containing 15% fetal bovine serum, 100 U/mL penicillin, and 100 U/mL streptomycin at 37 °C with 5% CO_2_. After 48 h, the cell morphology was observed and photographed under an inverted microscope.

Immunofluorescence staining was performed to identify granulosa cells specifically expressing follicle stimulating hormone receptor (FSHR). The cells were immunostained with primary antibody to FSHR (PA5-99424, 1:100, Invitrogen) overnight at 4 °C. Following three rinses with PBS, the cells were incubated with AlexFluor®488-labeled secondary antibody (1:1000, Jackson, USA) at 4 °C for 1 h without light exposure, and stained with DAPI (C1002, Beyotime) for 5 min. Finally, the cells were observed under a confocal microscope (OLYMPUS, IX73).

Cells were transduced with lentiviral plasmids carrying Vector, PKP3, short hairpin RNA (sh)-NC, sh-PKP3-1, sh-PKP3-2, sh-ERCC1-1, and sh-ERCC1-2. The cells in the logarithmic growth phase were digested with trypsin and dispersed into cell suspension (5 × 10^4^ cells/mL), which was then seeded in a 6-well plate (2 mL per well) and cultured overnight at 37 °C. After 48 h of transduction, the green fluorescent protein expression efficiency was observed by a fluorescence microscope. The shRNA sequence was designed by Life Technologies and synthesized by Shanghai GenePharma Co., Ltd. (Shanghai, China), with the sequence shown in Table S2.

For cell treatment, 0.5 µM DNA methyltransferase inhibitor 5-Aza-CdR (S1200, Selleck Chemicals, Houston, TX, USA) or CFDTW was applied to treat rat ovarian granulosa cells for 24 h.

To verify that the CFDTW/PKP3/MAPK/ERCC1 signaling axis promotes ovarian granulosa cell proliferation and inhibits its apoptosis, we simultaneously interfered ovarian granulosa cells with overexpression of PKP3 and knockdown of ERCC1. The detailed grouping: Vector + sh-NC, Vector + sh-NC + CFDTW, PKP3 + sh-NC + CFDTW and PKP3 + sh-ERCC1 + CFDTW.

### Cell counting kit-8 (CCK-8) assay

Cells were seeded into 96-well plates at a density of 2 × 10^3^ cells/well. At 12, 24, 48 and 72 h of culture, the cells in each well were incubated with 10 µL CCK-8 solution at 37 °C for 1–2 h. The optical density (OD) value of each well was measured using a microplate (Tecan, Switzerland) at 450 nm.

### RNA extraction and quantification

Total RNA was extracted from tissues using TRIzol reagent (16096020, Invitrogen). Next, the extracted RNA was reversely transcribed into complementary DNA using Reverse Transcription Kit (RR047A, Takara, Japan). RT-qPCR was then performed using TaqMan Gene Expression Assays (Applied Biosystems, Foster City, CA, USA) on an ABI 7500 instrument (Applied Biosystems). The primers for PKP3 and ERCC1 were designed on NCBI and their sequences are shown in Table S3. The fold changes were calculated by means of the 2^−∆∆Ct^ method.

### Western blot analysis

Total protein extracts were separated by polyacrylamide gel electrophoresis and transferred onto polyvinylidene fluoride membranes (IPVH85R, Millipore, Germany). The membrane was blocked with 5% bovine serum albumin and probed overnight at 4℃ with primary antibodies against PKP3 (18338-1-AP, 1:1000, Proteintech), ERCC1 (PA5-79217, 1:1000, Invitrogen), Bcl-2 (ab194583, 1:1000, Abcam, Cambridge, UK), Bax (ab32503, 1:1000, Abcam), Total-Cap3 (ab184787, 1:1000, Abcam), Cleaved Caspase-3 (9661, 1:1000, Cell Signaling Technologies [CST], Beverly, MA, USA), ERK1/2 (ab184699, 1:1000, Abcam), phospho-ERK1/2 (ab201015, 1:400, Abcam), p38 (14064-1-AP, 1:1000, Proteintech), phospho-p38 (4511, 1:1000, CST), and β-actin (ab8226, 1:5000, Abcam, serving as the loading control). The membrane was re-probed with the horseradish peroxidase (HRP)-conjugated secondary goat anti-rabbit IgG (ab6721, Abcam) or goat anti-mouse IgG (ab6789, Abcam) at room temperature for 1 h. The immunocomplexes on the membrane were visualized using luminescent liquid (1705062, Bio-Rad, California, USA) on Image Quant LAS 4000 C gel imager (GE Healthcare, Chicago, Illinois, USA) and band intensities were assayed using Image J software (National Institutes of Health).

### Flow cytometry

Annexin V-fluorescein isothiocyanate/propidium iodide (FITC/PI) double staining was used to examine apoptosis of ovarian granulosa cells. The cells were seeded in a 6-well plate at a density of 2 × 10^5^ cells/well, resuspended in 500 µL binding buffer on the basis of the instructions of BD Apoptosis Detection Kit (556547, BD Biosciences, Franklin Lakes, NJ, USA), and added with 5 µL FITC and 5 µL PI in the dark, followed by mixture and incubation for 15 min. Finally, a flow cytometer (FACSCalibur; BD, San Jose, CA, USA) was employed to detect cell apoptosis.

Cell cycle was analyzed. Briefly, cells were trypsinized, harvested, fixed overnight in 75% ethanol at -20 °C, and resuspended with 500 µL PI/RNase staining buffer (BD). After 15-min of incubation, the cells were filtered with a 400-mesh sieve. Cell cycle analysis was conducted using a flow cytometry.

### Statistical analysis

All data were assayed employing Graphpad Prism (Graphpad software, La Jolla, CA, USA). *p* value was determined by performing a two-tailed *t* test on independent samples. The measurement data were described as mean ± standard deviation. Data comparison between two groups was assayed by unpaired *t*-test. Differences among multiple groups were statistically analyzed employing one-way analysis of variance. *p* < 0.05 was deemed as statistically significant.

## Results

### CFDTW downregulates PKP3 by promoting the methylation of PKP3 promoter in PCOS patients

**Fig. 1:**
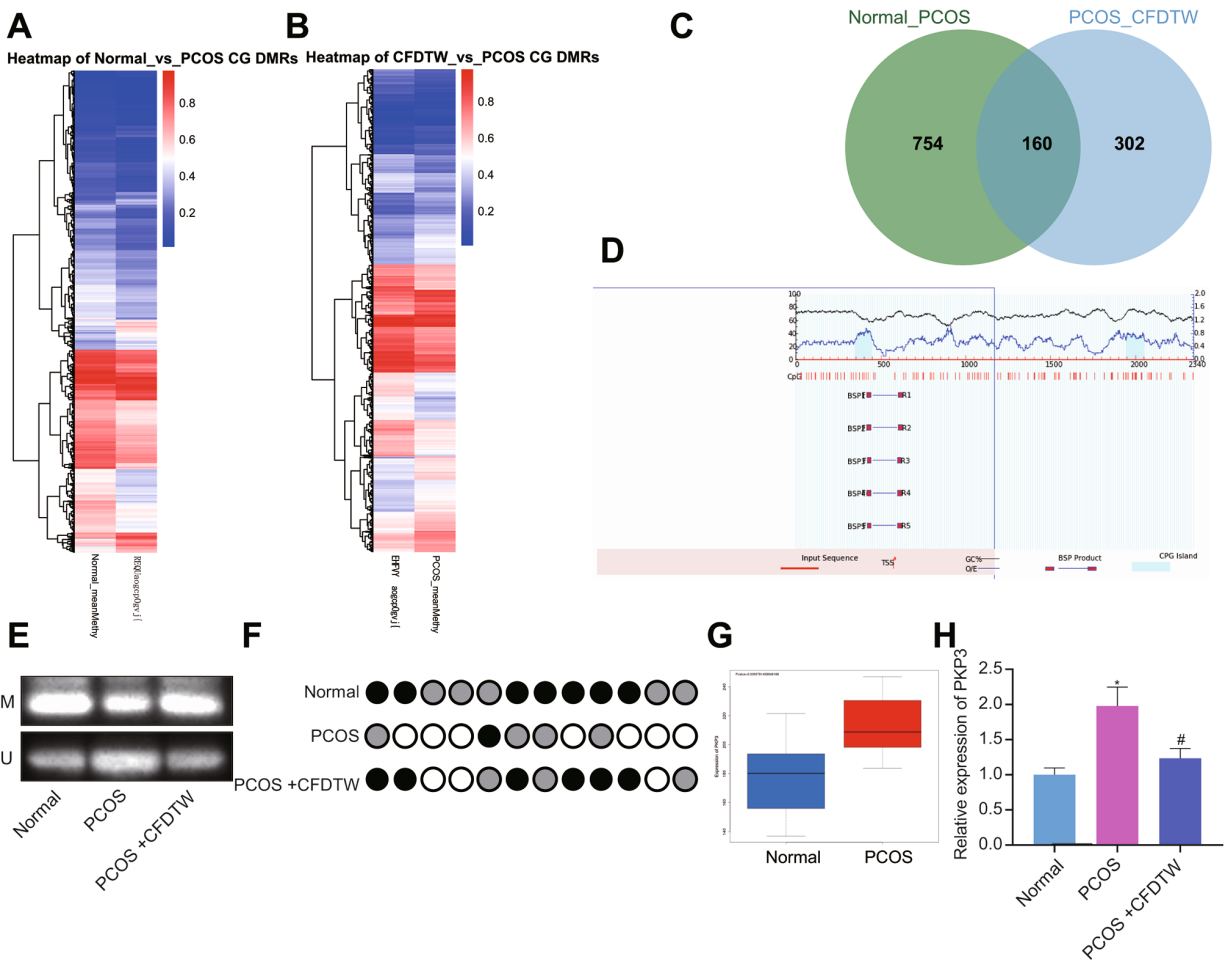
CFDTW reduces the expression of PKP3 by augmenting the PKP3 promoter methylation in PCOS patients. **A**, A clustering heat map of methylation levels in ovarian granulosa cells from healthy controls (n = 6) and PCOS patients with PDS (n = 12) based on RRBS data. **B**, A clustering heat map of methylation levels in ovarian granulosa cells from PCOS patients with PDS (n = 6) and CFDTW-treated PCOS patients with PDS (n = 6) based on RRBS data. The abscissa represents the group and the ordinate represents the clustering effect of the methylation level value, from blue to red representing the increasing methylation level. **C**, Venn diagram of differential methylation analysis results between healthy controls and PCOS patients, PCOS patients and CFDTW-treated PCOS patients. **D**, Schematic diagram of the CpG islands of the human PKP3 gene promoter. **E**, The methylation degree of PKP3 gene promoter in ovarian granulosa cell samples isolated from clinical patients determined by MSP assay. **F**, The methylation status of CpG islands in PKP3 gene promoter in ovarian granulosa cell samples isolated from clinical patients determined by BSP assay. The color of the circle at each CpG site represents the percentage of methylation. **G**, A box plot of PKP3 gene expression in PCOS-related GSE8157 dataset. Blue represents control samples (n = 13), and red represents PCOS samples (n = 10). **H**, mRNA expression of PKP3 determined by RT-qPCR in ovarian granulosa cell samples isolated from clinical patients. * *p* < 0.05 vs. healthy controls. # *p* < 0.05 vs. PCOS patients with PDS

Ovarian granulosa cells were collected from healthy controls, PCOS patients with PDS, and CFDTW-treated PCOS patients with PDS for gene DNA methylation sequencing. Based on the RRBS data, the differential methylation analysis results in the three groups were obtained (Fig. 1A, B). The overlapping genelist was then identified, which revealed 160 key factors affecting PCOS and CFDTW treatment (Fig. 1C).

MethPrimer website analysis showed the presence of CpG islands in the human PKP3 promoter sequence (Fig. 1D). In addition, MSP and BSP assays revealed that PKP3 promoter methylation was diminished in ovarian granulosa cells of PCOS patients while it was increased following CFDTW treatment (Fig. 1E, F). Meanwhile, differential analysis of the GSE8157 dataset exhibited that PKP3 was highly expressed in PCOS samples (Fig. 1G). Further RT-qPCR results presented higher expression of PKP3 mRNA in ovarian granulosa cells in the PCOS patients than that in the healthy controls while CFDTW treatment inhibited PKP3 mRNA expression (Fig. 1H). These results indicate that CFDTW may inhibit PKP3 expression by promoting methylation of the PKP3 promoter.

### CFDTW downregulates PKP3 by promoting the methylation of PKP3 promoter in PCOS rats

**Fig. 2:**
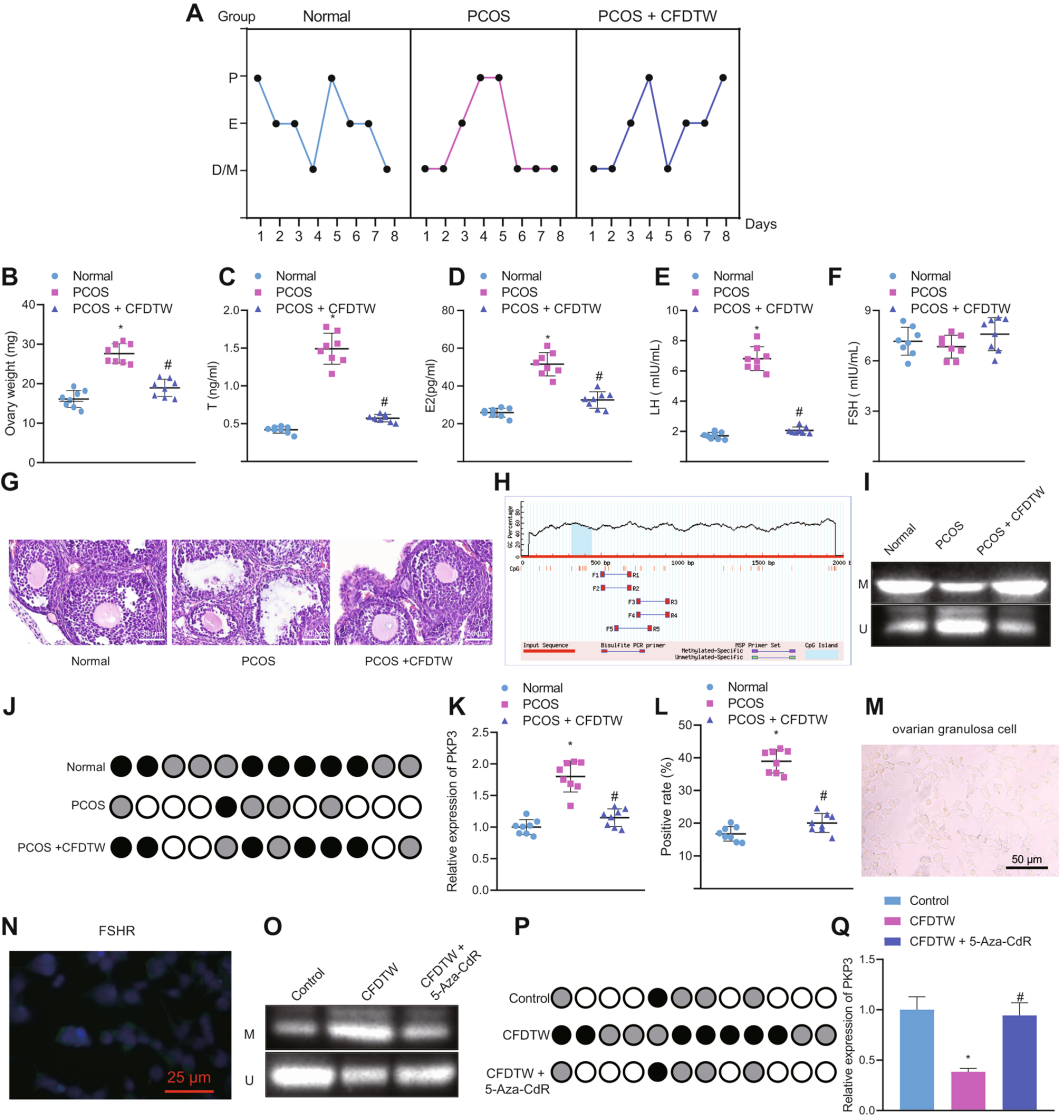
CFDTW reduces the expression of PKP3 by augmenting the PKP3 promoter methylation in PCOS rats. Normal rats served as controls and PCOS rats were further treated with CFDTW. **A**, Rat estrous cycle (P: proestrus; E: estrus; M: post-estrus; D: during estrus). **B**, Rat ovarian weight. **C**, Serum levels of T in rats. **D**, Serum levels of E2 in rats. **E**, Serum levels of LH in rats. **F**, Serum levels of FSH in rats. **G**, HE staining images of rat ovarian tissues, scale bar 50 μm. **H**, Schematic diagram of CpG islands in the rat PKP3 gene promoter. **I**, The methylation degree of the PKP3 promoter in rat ovarian tissues determined by MSP. **J**, Methylation status of CpG islands in the PKP3 promoter in rat ovarian tissues determined by BSP. The color of the circle at each CpG site represents the percentage of methylation. **K-L**, RT-qPCR detection (K) and immunohistochemistry analysis (L) of PKP3 expression in the rat ovarian tissue. **M**, Representative microscopic views of the ovarian granulosa cells isolated from rats under an inverted microscope, scale bar 50 μm. **N**, Immunofluorescence staining analysis of FSHR expression (green fluorescence) in the ovarian granulosa cells isolated from rats, scale bar 25 μm. **O**, The methylation degree of the PKP3 promoter in rat ovarian granulosa cells determined by MSP. **P**, Methylation status of CpG islands in the PKP3 promoter in rat ovarian granulosa cells determined by BSP. The color of the circle at each CpG site represents the percentage of methylation. **Q**, RT-qPCR detection of PKP3 expression in rat ovarian granulosa cells. * *p* < 0.05 vs. normal rats or control cells. # *p* < 0.05 vs. PCOS rats or those treated with CFDTW. n = 8 for rats following each treatment

Subcutaneous injection of DHEA was employed to establish a rat PCOS model. As shown in Fig. 2A-G, PCOS rats showed estrous cycle and reproductive hormone disorders, elevated serum levels of E2, T, and LH, and ovarian weight, reduced number of granulosa cells, as well as appearance of cystic follicles in the tissues but no alteration in FSH serum levels. The results demonstrated the successful construction of PCOS rat models.

In addition, CFDTW treatment restored the estrous cycle (Fig. 2A), reduced ovarian weight (Fig. 2B) and the serum levels of E2, T, and LH, but no alteration was seen in FSH serum level in PCOS rats (Fig. 2C-F). Meanwhile, the symptoms of polycystic changes in ovarian tissues were improved (Fig. 2G). Collectively, CFDTW can alleviate the symptoms of PCOS rats.

Analysis using the MethPrimer website revealed the CpG islands in the rat PKP3 promoter sequence (Fig. 2H). In addition, MSP and BSP assays showed that the methylation of the PKP3 promoter was reduced in the ovarian tissues of PCOS rats but CFDTW treatment led to opposite results (Fig. 2I, J). RT-qPCR and immunohistochemistry results illustrated an enhancement in PKP3 expression in the ovarian tissues of PCOS rats, which was reversed following CFDTW treatment (Fig. 2K, L).

Under an inverted microscope, the ovarian granulosa cells isolated from rats were spindle-shaped or polygonal (Fig. 2M) and expressed FSHR (Fig. 2N), indicating the successful isolation of rat ovarian granulosa cells. As depicted in Fig. 2O-Q, CFDTW treatment augmented the methylation level of PKP3 promoter while decreasing its expression. Conversely, 5-Aza-CdR abolished the effect of CFDTW on the methylation level of PKP3 promoter and its expression. The aforementioned data support that PKP3 may be highly expressed in PCOS rats, with hypomethylated promoter. CFDTW can inhibit PKP3 expression by promoting the methylation of PKP3 promoter.

### Silencing of PKP3 facilitates the proliferation of ovarian granulosa cells and inhibits their apoptosis

**Fig. 3:**
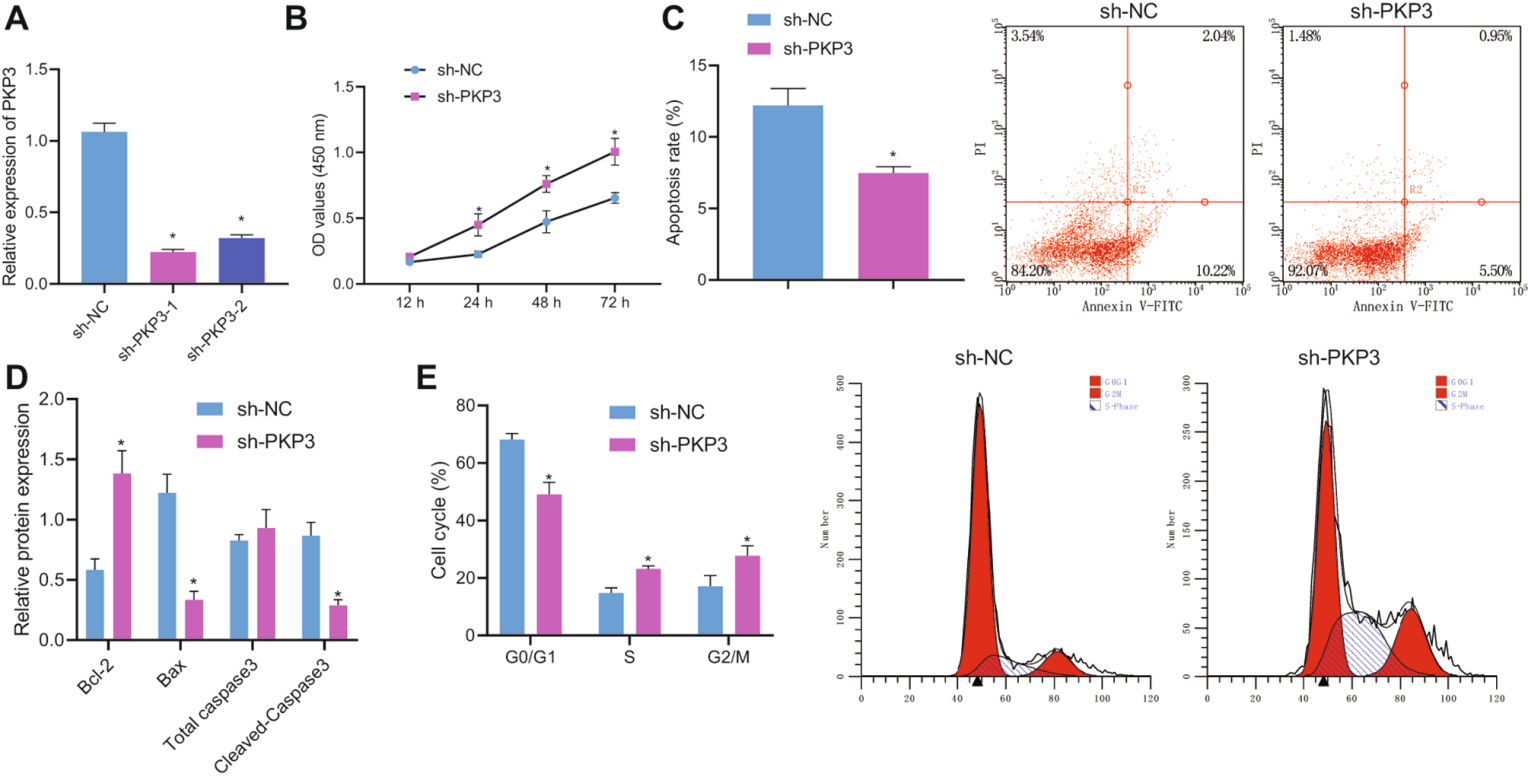
Silencing of PKP3 stimulates the proliferation of ovarian granulosa cells and represses their apoptosis. **A**, mRNA expression of PKP3 determined by RT-qPCR in ovarian granulosa cells treated with sh-PKP3-1 or sh-PKP3-2. B-C, Viability (**B**) and apoptosis (**C**) of ovarian granulosa cells with PKP3 knockdown measured by CCK-8 assay and flow cytometry. **D**, Expression of Bcl-2, Bax, and cleaved caspase-3 in ovarian granulosa cells with PKP3 knockdown determined by Western blot analysis. **E**, Cell cycle distribution of ovarian granulosa cells with PKP3 knockdown measured by flow cytometry. * *p* < 0.05, ** *p* < 0.01. The cell experiment was conducted three times independently

Next, the focus was to elucidate the role of PKP3 gene in PCOS. RT-qPCR results showed that sh-PKP3-1 had a better silencing efficiency (Fig. 3A) and was therefore chosen for subsequent experimentations. CCK-8 assay displayed ascending cell viability result in the presence of PKP3 knockdown (Fig. 3B). Flow cytometric analysis showed that the number of apoptotic cells was decreased following PKP3 knockdown (Fig. 3C). At the same time, PKP3 knockdown elevated expression of Bcl-2 and reduced that of Bax and cleaved caspase-3 (Fig. 3D). In addition, G0/G1 phase-arrested cells were reduced whereas S phase- and G2/M phase-arrested cells were increased in the absence of PKP3 (Fig. 3E). Therefore, PKP3 silencing can contribute to enhancement in the proliferation of ovarian granulosa cells and inhibition of their apoptosis.

### PKP3 upregulates ERCC1 by activating the MAPK pathway

**Fig. 4:**
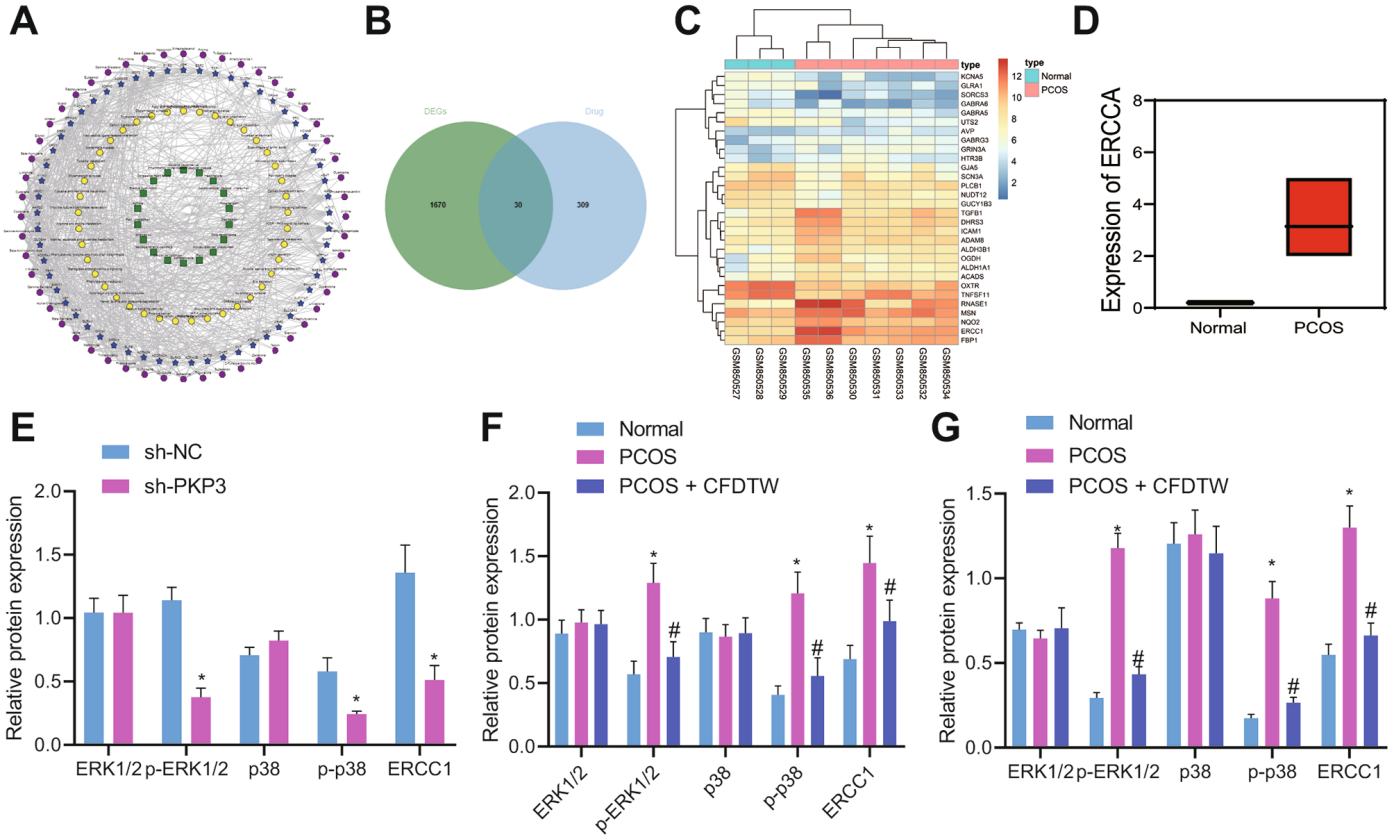
PKP3 enhances the expression of ERCC1 by activating the MAPK pathway. **A**, CFDTW component-potential target-pathway network diagram. **B**, Venn diagram of the differentially expressed genes in the PCOS samples in the GSE34526 dataset and CFDTW potential targets. **C**, An expression heat map of the intersection genes in the GSE34526 dataset. **D**, A box plot of ERCC1 gene expression in the GSE34526 dataset. Blue represents control samples (n = 3) and red represents PCOS samples (n = 7). **E**, ERCC1 protein expression and the extent of ERK1/2 and p38 phosphorylation in ovarian granulosa cells with PKP3 knockdown determined by Western blot analysis. **F**, ERCC1 protein expression and the extent of ERK1/2 and p38 phosphorylation in ovarian granulosa cells from PCOS patients with PDS or those treated with CFDTW determined by Western blot analysis. **G**, ERCC1 protein expression and the extent of ERK1/2 and p38 phosphorylation in ovarian tissues of PCOS rats (n = 8) or those treated with CFDTW (n = 8) determined by Western blot analysis. * *p* < 0.05. The cell experiment was conducted three times independently

Subsequently, we sought to dissect out the mechanism underlying the promoting effect of PKP3 on ovarian granulosa cell proliferation and inhibiting effect on cell apoptosis. Differential analysis on the PCOS-related GSE34526 dataset yielded 1700 differentially expressed genes in PCOS samples, of which, 30 genes were potential targets of CFDTW (Fig. 4A, B). Of these 30 genes, ERCC1 was found to be robustly induced in PCOS samples (Fig. 4C, D). Exiting literature has indicated that inhibiting the MAPK pathway can reduce ERCC1 expression [14]. Therefore, we speculate that CFDTW may inactivate the MAPK pathway by promoting methylation of the PKP3 promoter, thereby inhibiting ERCC1 expression.

Western blot analysis results demonstrated that PKP3 knockdown decreased ERCC1 protein expression and the extent of ERK1/2 and p38 phosphorylation in ovarian granulosa cells (Fig. 4E). Moreover, ERCC1 protein expression and the extent of ERK1/2 and p38 phosphorylation were higher in the ovarian granulosa cells of PCOS patients with PDS compared with healthy controls. However, CFDTW treatment led to contrary results (Fig. 4F). Similar results were obtained in the rat ovarian tissues (Fig. 4G). Taken together, PKP3 may upregulate ERCC1 by activating the MAPK pathway.

### CFDTW potentiates the proliferation of ovarian granulosa cells and inhibits their apoptosis by regulating the PKP3/MAPK/ERCC1 axis

**Fig. 5:**
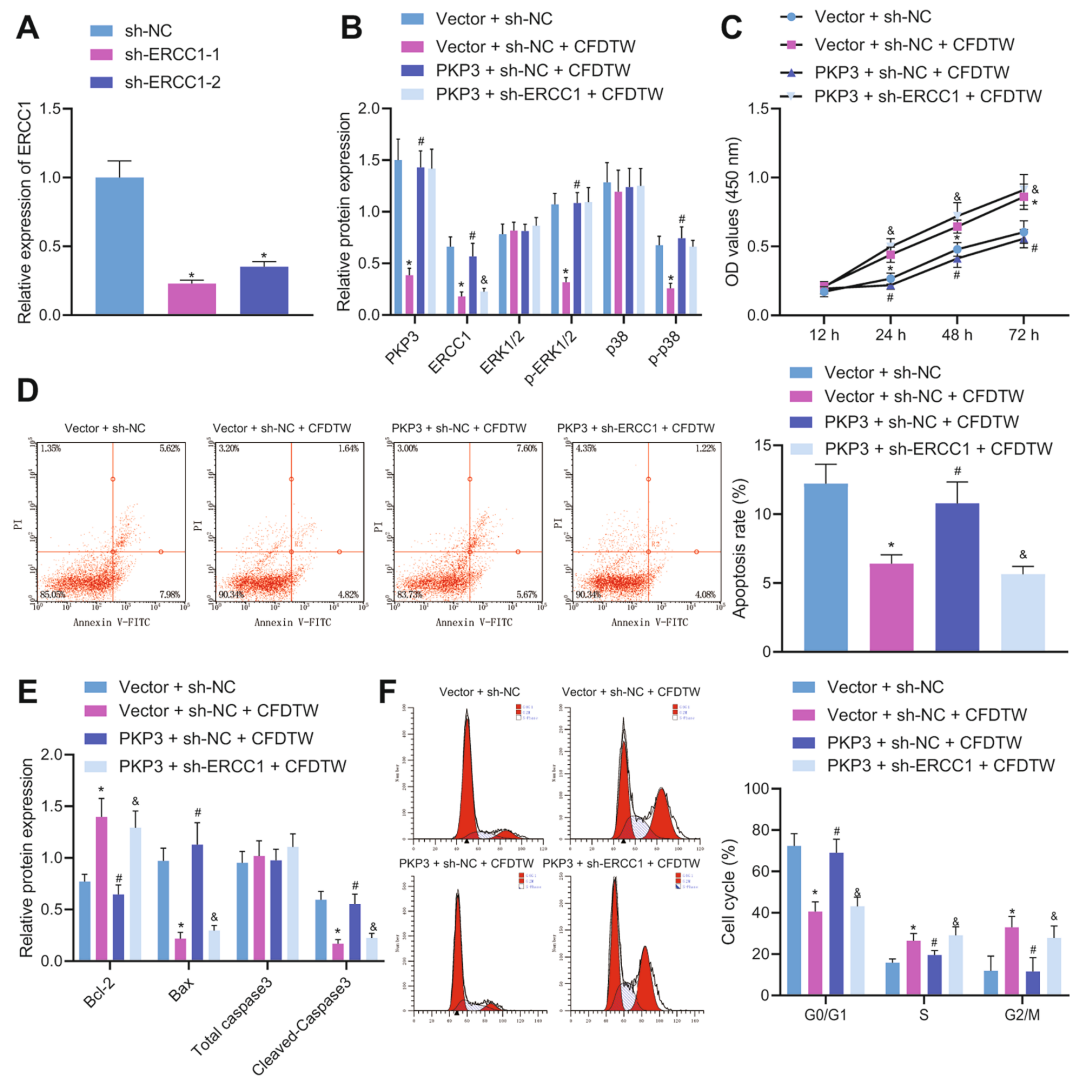
CFDTW stimulates the proliferation of ovarian granulosa cells and represses their apoptosis by regulating the PKP3/MAPK/ERCC1 axis. **A**, ERCC1 mRNA expression in ovarian granulosa cells treated with sh-ERCC1-1 or sh-ERCC1-2 determined by RT-qPCR. Ovarian granulosa cells were treated with Vector + sh-NC + CFDTW, PKP3 + sh-NC + CFDTW or PKP3 + sh-ERCC1 + CFDTW. **B**, ERCC1 protein expression and the extent of ERK1/2 and p38 phosphorylation in ovarian granulosa cells determined by Western blot analysis. C-D, Viability (**C**) and apoptosis (**D**) of ovarian granulosa cells measured by CCK-8 assay and flow cytometry. **E**, Expression of Bcl-2, Bax, and cleaved caspase-3 in ovarian granulosa cells determined by Western blot analysis. **F**, Cell cycle distribution of ovarian granulosa cells measured by flow cytometry. * *p* < 0.05. The cell experiment was conducted three times independently

The aforementioned results allowed us to characterize the effect of CFDTW on ovarian granulosa cells *via* regulation of the PKP3/MAPK/ERCC1 signaling axis. RT-qPCR confirmed that sh-ERCC1-1 had the superior silencing efficiency (Fig. 5A) and was thus selected for follow-up experimentations. Western blot analysis results suggested a decline in the ERCC1 protein expression and the extent of ERK1/2 and p38 phosphorylation in ovarian granulosa cells treated with Vector + sh-NC + CFDTW than those treated with Vector + sh-NC. In contrast, further overexpression of PKP3 caused an opposite result. Additionally, ERCC1 protein expression was noted to be diminished following treatment with PKP3 + sh-ERCC1 + CFDTW while PKP3 protein expression and the extent of ERK1/2 and p38 phosphorylation exhibited no alterations (Fig. 5B).

As shown in Fig. 5C, D, CFDTW treatment led to increased cell viability and reduced apoptosis rate; while combined treatment with PKP3 and CFDTW led to lower cell viability and higher apoptosis rate than individual CFDTW treatment. Relative to combined treatment with PKP3 and CFDTW, further sh-ERCC1 treatment caused enhanced cell viability and reduced apoptosis rate. Additionally, treatment with PKP3 + sh-NC + CFDTW reduced Bcl-2 expression and elevated that of Bax and cleaved caspase-3, while opposite results were noted following treatment with Vector + sh-NC + CFDTW or PKP3 + sh-ERCC1 + CFDTW (Fig. 5E). In addition, higher G0/G1 phase-arrested cells and lower S phase- and G2/M phase-arrested cells were noted in response to treatment with PKP3 + sh-NC + CFDTW than with Vector + sh-NC + CFDTW. Conversely, G0/G1 phase-arrested cells were decreased while S phase- and G2/M phase-arrested cells were increased in the presence of Vector + sh-NC + CFDTW or PKP3 + sh-ERCC1 + CFDTW (Fig. 5F). Taken together, CFDTW can inactivate the MAPK pathway by inhibiting PKP3 expression, and downregulate ERCC1, thereby promoting the proliferation of ovarian granulosa cells and inhibiting their apoptosis.

## Discussion

The findings from our work pinpointed the promoting property of CFDTW in the proliferation of ovarian granulosa cells and inhibiting effect on the ensuing progression of PCOS with PDS *via* disruption of PKP3-mediated activation of MAPK pathway and ERCC1 expression.

DNA methylation has been highlighted to be a key epigenetic process which is essential for the regulation of gene expression; promoter-specific hypermethylation and concomitant gene silencing are associated with a broad array of diseases, including cancer [21, 22]. Our initial finding validated that CFDTW could reduce PKP3 expression by promoting the methylation of its promoter in PCOS. Recently published literature has indicated that altered methylation in genes involved in vital processes associated with follicular development potentially induces ovarian defects in women suffering from PCOS [23]. The current study, for the first time, demonstrated that silencing of PKP3 due to the increased promoter methylation could promote the proliferation of ovarian granulosa cells and inhibit their apoptosis, as evidenced by elevated expression of Bcl-2 and reduced that of Bax and cleaved caspase-3. Granulosa cells are well-known to be essential for normal follicular maturation as these cells can produce steroidal hormones and growth factors, and take a critical role in follicular atresia [24]. Elevated cleaved caspase-3/caspase-3 ratio but reduced Bcl-2/Bax ratio was detected in dehydroepiandrosterone-induced PCOS model, highly suggestive of increased apoptotic index [25]. Inhibition of proliferation yet promotion of apoptosis in ovarian granulosa cells have been confirmed to be responsible for the progression of PCOS [26, 27]. Overexpression of PKP3 has been documented to facilitate the malignant features of ovarian cancer cells [9]. However, the potential role of PKP3 in the cellular process of ovarian granulosa cells remains elusive, which warrants further investigation.

Further investigation revealed that PKP3 could upregulate ERCC1 by activating the MAPK pathway. In line with this, overexpression of PKP3 in ovarian cancer A2780 cells contributes to a significant activation of the MAPK pathway [9] Inactivating the MAPK pathway can reduce the insulin resistance, which represents an obstacle in the treatment efficacy of PCOS [28]. Meanwhile, activating p38 MAPK pathway is associated with the induction of G1/G0 phase-arrested ovarian granulosa cells [29]. Increased expression of ERCC1 can be achieved by activation of the p38 MAPK pathway [30]. A low expression of ERCC1 reflects a better chemosensitivity in ovarian cancer patients than a high expression [31]. Moreover, decreased expression of ERCC1 is strong related to improved progression free survival in patients with epithelial ovarian cancers [32]. The aforementioned results prove that inhibiting the PKP3/MAPK/ERCC1 axis can facilitate the development of therapeutic strategy against PCOS with PDS, but the correlation between PKP3 and ERCC1 still requires subsequent endeavors due to the limited data to support it.

Recent evidence has reported that CFDTW can effectively alleviate the symptoms of PCOS [6]. In addition, another study has also shown that CFDTW can serve as a PCOS treatment drug by decreasing the serum levels of TCHO, TG, LDL-c, LH, T, IL-1β, IL-6, and TNF-α, while increasing those of HDL-c in a dose-dependent manner [33], which is in agreement with our results that CFDTW treatment restored the estrous cycle, reduced the serum levels of T and LH in PCOS rats. Furthermore, in vitro data provided evidence that CFDTW potentiated the proliferation of ovarian granulosa cells and inhibited their apoptosis by regulating the PKP3/MAPK/ERCC1 axis, thus alleviating PCOS with PDS.

## Conclusion

**Fig. 6:**
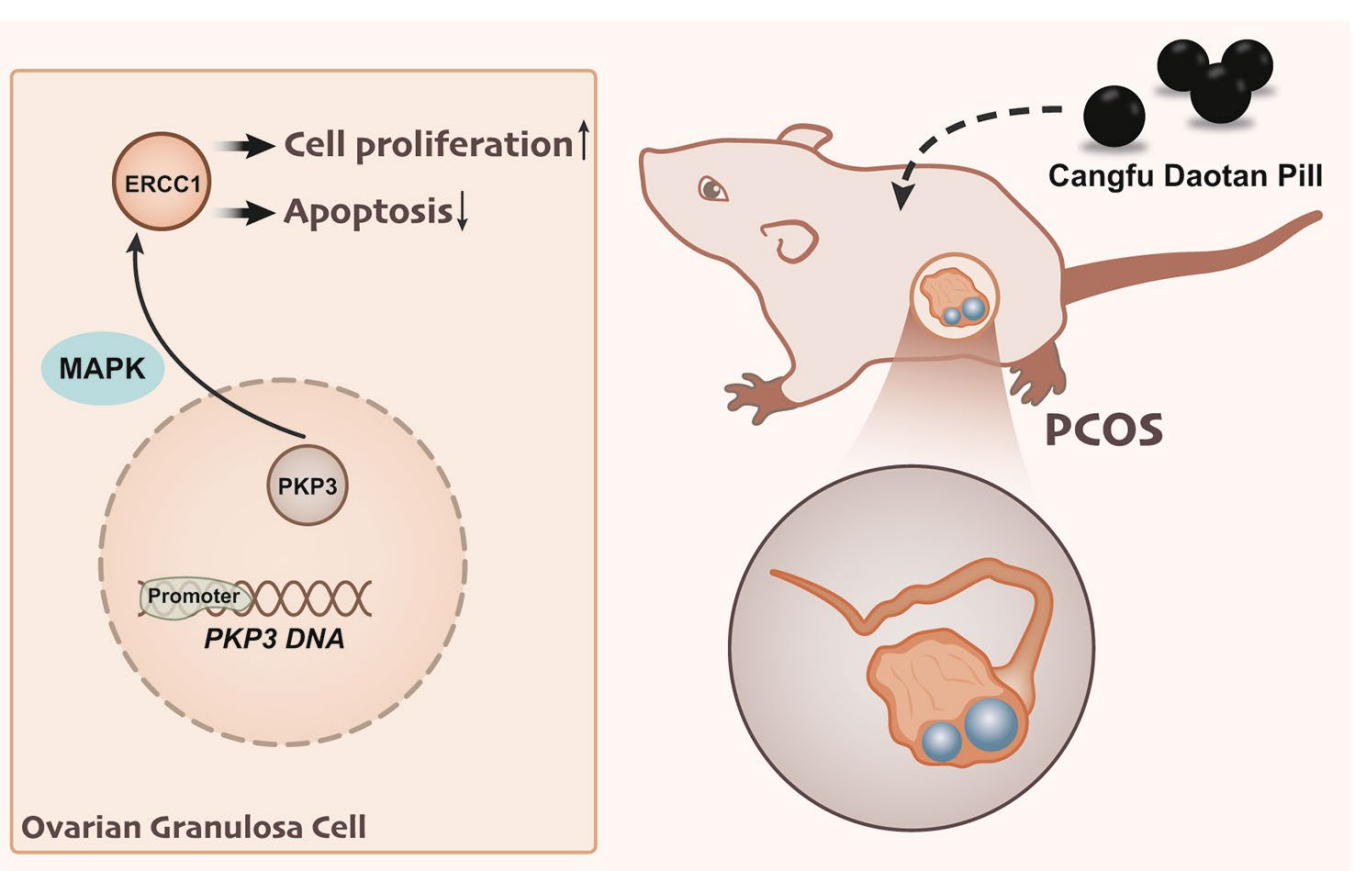
Schematic diagram of the mechanism by which CFDTW affects the PCOS with PDS. CFDTW promotes the methylation of PKP3 promoter in ovarian granulosa cells, leading to inhibition of PKP3 expression and decreased MAPK pathway activity, thereby inhibiting ERCC1 expression, promoting the proliferation of ovarian granulosa cells and inhibiting their apoptosis, and ultimately alleviating PCOS with PDS

In conclusion, CFDTW could potentially promote methylation of the PKP3 promoter, consequently inhibiting PKP3 expression, inactivating the MAPK pathway and downregulating ERCC1 expression. By this mechanism, the proliferation of ovarian granulosa cells was induced, their apoptosis was suppressed and resultant PCOS with PDS was arrested (Fig. 6). These results provide new insights into the mechanism underlying the progression of PCOS with PDS and might well aid intervention strategies of this disease in the future. Although this study has to some extent revealed the possible molecular mechanism of CFDTW in improving phlegm dampness type PCOS by regulating the PKP3/MAPK/ERCC1 signal axis, there are still the following limitations. Firstly, this study only collected ovarian granulosa cells from 12 patients with phlegm dampness type PCOS and 6 healthy women undergoing in vitro fertilization. The small sample size may not fully reflect the characteristics of the entire patient population. Future research should expand the sample size to increase the reliability and universality of the results. In addition, this study used rats as animal models to verify the therapeutic effects of CFDTW. Although rat models are commonly used as experimental animals in many studies, their physiological mechanisms may differ from those of humans. Future research may consider using other animal models or in vitro cell experiments to verify the therapeutic effect of CFDTW on phlegm dampness type PCOS, and to comprehensively understand the mechanism and potential targets of CFDTW in the treatment of phlegm dampness type PCOS.

## Electronic supplementary material

Below is the link to the electronic supplementary material.

Additional file 1: Figure S1. Identification of the estrous cycle of rats by the vaginal smear method, scale bar 50 μm. The red arrows represent nucleated epithelial cells, the green arrows represent keratinized squamous epithelial cells, and the black arrows represent white blood cells.

Additional file 2: Table S1-S3.

## Data Availability

The data that supports the findings of this study are available on request from the corresponding author upon reasonable request.
