# Supplementary material for: Cangfu Daotan Wan alleviates polycystic ovary syndrome with phlegm-dampness syndrome via disruption of the PKP3/ERCC1/MAPK axis
Source: J Ovarian Res. 2023 Jul 7;16:134. doi: 10.1186/s13048-023-01200-7 (PMC10327183; doi:10.1186/s13048-023-01200-7)
Supplement: Supplementary file 2 — Additional file 2: Table S1-S3. [file 13048_2023_1200_MOESM2_ESM.docx]

**Table S1** Primer sequences for MSP and BSP

| Gene | Sequence |
| --- | --- |
| PKP3 (human) | MF: 5’-TTTTAATTTTGATTCGGTTTTTACG-3’ |
|  | MR: 5’-AACAACCTCTAAACTTCTCTCACGA-3’ |
|  | UF: 5’-TTTAATTTTGATTTGGTTTTTATGT-3’ |
|  | UR: 5’-ACAACCTCTAAACTTCTCTCACAAA-3’ |
| PKP3 (rat) | MF: 5’-GATTAGGGTACGATTTGGATAGC-3’ |
|  | MR: 5’-ACTCTAAACAAAAACTTAAAACGCA-3’ |
|  | UF: 5’-ATTAGGGTATGATTTGGATAGTGG-3’ |
|  | UR: 5’-ACTCTAAACAAAAACTTAAAACACA-3’ |
| PKP3 (human) | Forward: 5’-TTAGTTTGGTTTTTGTAGGGTTTTA-3’ |
|  | Reverse: 5’-AAATATCCAAAACTCCATCCCTAAC-3’ |
| PKP3 (rat) | Forward: 5’-GGGTTTTTTTAAGGGTATTGAGAG-3’ |
|  | Reverse: 5’-AACCCTCCTAACCTCCCTAATTAAT-3’ |

MF, methylated forward; MR, methylated reverse; UF, unmethylated forward; UR, unmethylated reverse.

**Table S2** shRNA primer sequences

| shRNA | Sequence (5'-3') |
| --- | --- |
| sh-NC | GATCGTACTCACATCCACACT |
| sh-PKP3-1 | GCAGTGGCTTTGATGACATTG |
| sh-PKP3-2 | GCAGCGCTGTGAACTAAATCG |
| sh-ERCC1-1 | GCTCTTAAGGAGCTGGCTAAG |
| sh-ERCC1-2 | GCTGGAACAGAACTTCCTATC |

**Table S3** Primer sequences for RT-qPCR

| Gene | Sequence |
| --- | --- |
| PKP3 (rat) | Forward: 5’-GCGCTGTGAACTAAATCGGC-3’ |
|  | Reverse: 5’-GTTTCGGATGAGGCCAGTCA-3’ |
| ERCC1 (rat) | Forward: 5’-GACATACGCAGAGTACGCCA-3’ |
|  | Reverse: 5’-ATCAGGAGTCACCTCACCGA-3’ |
| GAPDH (rat) | Forward: 5’-GGCACAGTCAAGGCTGAGAAT -3’ |
|  | Reverse: 5’-ATGGTGGTGAAGACGCCAGTA-3’ |
